# Foxo3a-dependent Bim transcription protects mice from a high fat diet via inhibition of activation of the NLRP3 inflammasome by facilitating autophagy flux in Kupffer cells

**DOI:** 10.18632/oncotarget.15946

**Published:** 2017-03-06

**Authors:** Yan Liu, Wenfeng Zhang, Xiaoling Wu, Jian-Ping Gong

**Affiliations:** ^1^ Department of Digestive System, Second Affiliated Hospital of Chongqing Medical University, Chongqing, 400010, P.R. China; ^2^ Chongqing Key Laboratory of Hepatobiliary Surgery and Department of Hepatobiliary Surgery, Second Affiliated Hospital of Chongqing Medical University, Chongqing, 400010, P.R. China; ^3^ Department of Gastroenterology, The Fifth People's Hospital of Chengdu, Chengdu, Sichuan, 611130, P.R. China

**Keywords:** Foxo3a, autophagy, NLRP3 inflammasome, Bim, NAFLD

## Abstract

**Background:**

The role of Foxo3a in the regulation of autophagy flux and activation of the NLRP3 inflammasome in KCs suffering from HFD conditions is unknown.

**Results:**

Up-regulation of Foxo3a restored autophagy flux and dampened the activation of the NLRP3 inflammasome in KCs stimulated with PA and LPS. In contrast, down-regulation of Foxo3a increased blockage of autophagy flux and promoted NLRP3 inflammasome activation. Additionally, mRNA levels of Bim were significantly changed with the alteration of Foxo3a in KCs under PA and LPS stimulation among foxo3a targeted genes. Overexpression of Bim restored autophagy influx and attenuated NLRP3 inflammasome pathway activation. In addition, autophagy formation was restored, and activation of NLRP3 inflammasome was inhibited in KCs isolated from mice treated with Iturin A and fed with a HFD.

**Materials and methods:**

Autophagy flux in KCs and activation levels of NLRP3 inflammasome were evaluated after altering the expression of Foxo3a in KCs before stimulation with PA and LPS. Additionally, various target genes of Foxo3a were measured in KCs pretreated with an agonist (Iturin A) or inhibitor (SC97) of Foxo3a after KCs stimulation with PA and LPS in order to hunt for targets of Foxo3a. Activation levels of NLRP3 inflammasome in isolated KCs, as well as autophagy flux, were measured after mice were treated with Iturin A and fed with a HFD for 16 weeks.

**Conclusions:**

Foxo3a restores autophagy flux and attenuates the activation of the NLRP3 inflammasome by promoting the transcription of Bim, suggesting a potential therapeutic target in NAFLD and other obesity-related diseases.

## INTRODUCTION

Non-alcoholic fatty liver disease (NAFLD) is an increasing hepatic disease with a worldwide distribution [1]. Pathological processes in NAFLD include excessive accumulation of fat in the liver tissue as well as long-lasting and low grade inflammation, which eventually causes steatosis or even more malignant changes [2–3]. Increasing studies have confirmed that liver resident macrophages, Kupffer cells (KCs), mediate inflammation that aggravates the pathological processes of NAFLD [4–5]. The number of KCs is not changed in liver tissue bordering regions of steatohepatitis; however, fat-laden KCs accelerate inflammatory necrosis via altering their polarization state and activating the myeloid differentiation molecular 88 (MYD88)-dependent Toll-like receptor 4 (TLR4) signaling pathway [6]. In addition, free fatty acid and lipopolysaccharide (LPS) translocation from the gut leads to oxidative stress and stress in the endoplasmic reticulum, which causes production of harmful reactive oxygen species (ROS) and mitochondrial DNA (mtDNA) oxidative damage [7]. Activation of the TLR4 pathway and ROS damage both cue assembly and activation of the nucleotide oligomerization domain (NOD)-like receptor family pyrin domain containing 3 (NLRP3) inflammasome, which makes caspase-1 self-cleave to promote the secretion of mature interleukin (IL)-1β and IL-18 [8].

The NLRP3 inflammasome activated pathway is well described in macrophages suffering from foreign microbial infection or endogenous LPS stimulation [9–11]. The classical pathways of the NLRP3 inflammasome are activated by pathogen-associated molecular patterns (PAMPs) and danger-associated molecular patterns (DAMPs), further promoting TLR4/nuclear factor κb (NF-κb) signaling to produce precursors of IL-1β and IL-18 [9–10]. The latter are cleaved by the NLRP3 inflammasome/apoptosis associated speck-like protein (ASC)/pro-caspase-1 complex and become entire activated states [9–10]. Another pathway of NLRP3 inflammasome activation is independent of TLR4; intracellular caspase-11 is directly activated by LPS, which promotes gasdermin D release and IL-1β and IL-18 maturation [11–12]. The NLRP3 inflammasome pathway also plays a critical role in cell apoptosis, autophagy and metabolism [13]. Thus, inhibition of NLRP3 inflammasome activation in KCs may be an effective way to limit the shifting of NAFLD to non-alcoholic steatohepatitis (NASH).

NAFLD is both a process and outcome of persistent inflammation and disorders of lipid metabolism [14]. Recently, Liu et al. asserted that macrophage autophagy is impaired in high fat diet (HFD)-fed mice, with proinflammatory macrophage polarization [15]. Autophagy is a highly conserved biological phenomenon that maintains homeostasis and adapts to changes in the microenvironment [16]. Autophagy is initiated when the cell's sensory receptors perceive harmful intracellular and extracellular factors such as starvation, ROS, toxic lipid, LPS, and inflammation mediators [17]. NLRP3 inflammasome activation also initiates autophagy in macrophages, and loss of autophagy in macrophages enhances the activation of NLRP3 inflammasome inflammatory pathways and down-regulates the stability of mitochondria via excessive ROS generation [18]. However, formation of autolysosomes in macrophages is blocked when the mice are fed a HFD [15]. This suggests that autophagy restoration can restrain the damage caused by NLRP3 inflammasome activation in NAFLD.

Fork O3A protein (Foxo3a) is a member of the Fork frame transcription factor family, and it has been asserted that AMP-activated protein kinase (AMPK)-dependent phosphorylation of Foxo3a represses transcription of S phase kinase-associated protein 2 (SKP2), which subsequently initiates autophagy formation in nutrient-deprived conditions [19]. Conversely, liraglutide protects mice from a HFD by promoting autophagy through the silent information regulator 1 (SIRT1)/SIRT3-Foxo3a pathway [20]. Thus, Foxo3a coordinates a protective effect of the autophagy process in over-nutrition conditions. Additionally, Foxo3a interacts with NF-κB in the cytosol to prevent Foxo3a degradation and NF-κB nuclear translocation [21]. In this way, proinflammatory signaling pathways are efficiently inactivated. Additionally, Foxo3a phosphorylates inhibitor of NF-κB α (IκB-α) to activate the NF-κB pathway when macrophages are stimulated by LPS [22]. The status of NF-κB directly impacts the activation of the NLRP3 inflammasome pathway as mentioned above. Presumably, Foxo3a bridges autophagy program initiation and NLRP3 inflammasome pathway activation in KCs suffering from excessive free fatty acid (FFA) and LPS. However, little is known about the effects and patterns of Foxo3a in KCs regarding the regulation of the autophagy program and the NLRP3 inflammasome pathway in NAFLD. Therefore, we investigated and have found an autophagy flux change, as well as an activation level change of the NLRP3 inflammasome pathway, after altering the expression of Foxo3a in KCs under FFA and LPS stimulation *in vitro*. Additionally, we have found that mRNA levels of Bim are significantly changed with the alteration of Foxo3a in KCs under FFA and LPS stimulation among Foxo3a targeted transcription genes. Furthermore, overexpression of Bim, which leads to up-regulation of Beclin 1 in KCs that are pretreated with a Foxo3a inhibitor restores autophagy influx and attenuates NLRP3 inflammasome pathway activation. Autophagy flux and NLRP3 inflammasome status in KCs were also evaluated in mice fed with a HFD. Liver steatosis and insulin tolerance in the HFD-mice treated with a Foxo3a agonist were milder than in mice fed a HFD alone. In addition, autophagy flux was increased and NLRP3 inflammasome activation was dampened in KCs isolated from HFD-mice treated with a Foxo3a agonist. Therefore, we have illuminated another pattern of Foxo3a function in KCs for regulating the autophagy program and the NLRP3 inflammasome pathway in NAFLD.

## RESULTS

### Combination effects of PA and LPS on activation of the NLRP3 inflammasome, autophagy flux and Foxo3a expression in KCs

**Figure 1:**
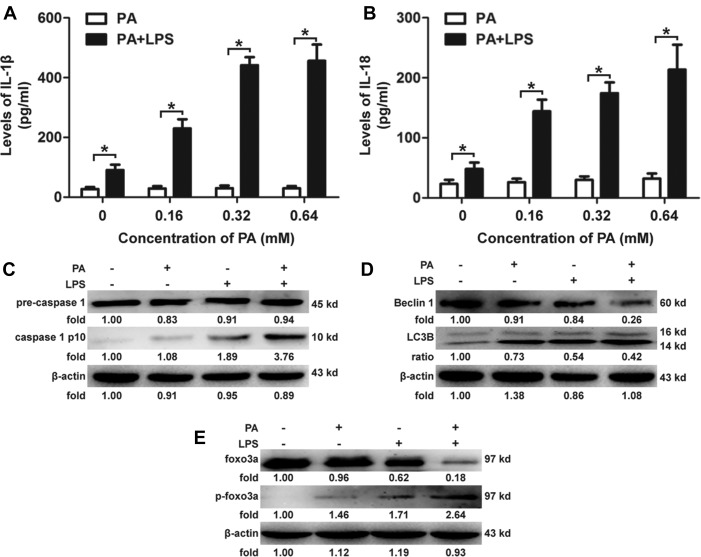
Combination effects of PA and LPS on KCs (**A** and **B**): KCs were treated with PA (0, 0.16, 0.32, 0.64 mM) for 12 h after stimulation with LPS (100 ng/ml) for 1 h. Levels of IL-1β were significantly up-regulated in KCs treated with PA and LPS compared to those treated with PA alone. Dose-dependent effects were observed in combination stimulation of PA and LPS. *p < 0.05. (**C–E**): KCs were treated with PA (0.5 mM) for 12 h after stimulation with LPS (100 ng/ml) for 1 h, and increased caspase-1 p10 separated from pro-caspase-1 was observed in KCs treated with PA and LPS compared to those treated with PA alone; protein expression of Beclin 1, as well as the ratio of LC3 II and I, was inhibited by PA and LPS compared to those treated with PA alone; phosphorylation levels of Foxo3a were significantly up-regulated compared to levels in KCs treated with PA alone, whereas protein levels of Foxo3a were significantly down-regulated in the same groups.

KCs were treated with palmitic acid (PA) (0, 0.16, 0.32, 0.64 mM) for 12 h after stimulation with LPS (100 ng/ml) for 1 h. As representative cytokines of NLRP3 inflammasome activation, supernatant levels of IL-1β and IL-18 were not changed when KCs were treated with PA alone (Figure 1A and 1B). Levels of IL-1β and IL-18 in KCs treated with LPS and PA were significantly higher than in KCs treated with PA alone (Figure 1A and 1B). Caspase-1 separated from pro-caspase-1 was observed in KCs treated with PA and LPS (Figure 1C). Protein expression of Beclin 1, as well as the ratio of anti-Light Chain 3 (LC3) II and I, were inhibited by PA and LPS (Figure 1D). Furthermore, phosphorylation levels of Foxo3a were significantly up-regulated in KCs treated with LPS and PA than in KCs treated with PA or LPS alone; meanwhile, protein levels of Foxo3a were significantly down-regulated in KCs treated with LPS and PA than in KCs treated with PA or LPS alone (Figure 1E). ROS and LC3 punctae were observed to significantly increase in KCs treated with PA and LPS (Supplementary Figure 3A and 3B). The upstream regulators of Foxo3a, such as AMPK and phosphatidyl inositol 3-kinase (PI3K)/AKT were also investigated in KCs treated with PA and LPS. The phosphorylation levels of PI3K/AKT were significantly higher in KCs treated with LPS and PA than in KCs treated with PA or LPS alone. Thus, the data show that double hits (i.e., LPS and PA) activate the NLRP3 inflammasome pathway, block autophagy flux and can alter the status of Foxo3a within the nucleus (Supplementary Figure 3C). These results concerning the activation of the NLRP3 inflammasome pathway were consistent with previous reports [15, 23–34], despite PA alone previously being reported as an activator of the NLRP3 inflammasome, leading to increased levels of IL-1β and IL-18 in macrophages. However, to determine whether the autophagy flux would be restored and the activation of the NLRP3 inflammasome pathway would be attenuated after recovery of the expression of Foxo3a in KCs, we continued to investigate in the following experiments.

### Effects of Foxo3a on the autophagy flux and the activation of the NLRP3 inflammasome in KCs after stimulation with PA and LPS

**Figure 2:**
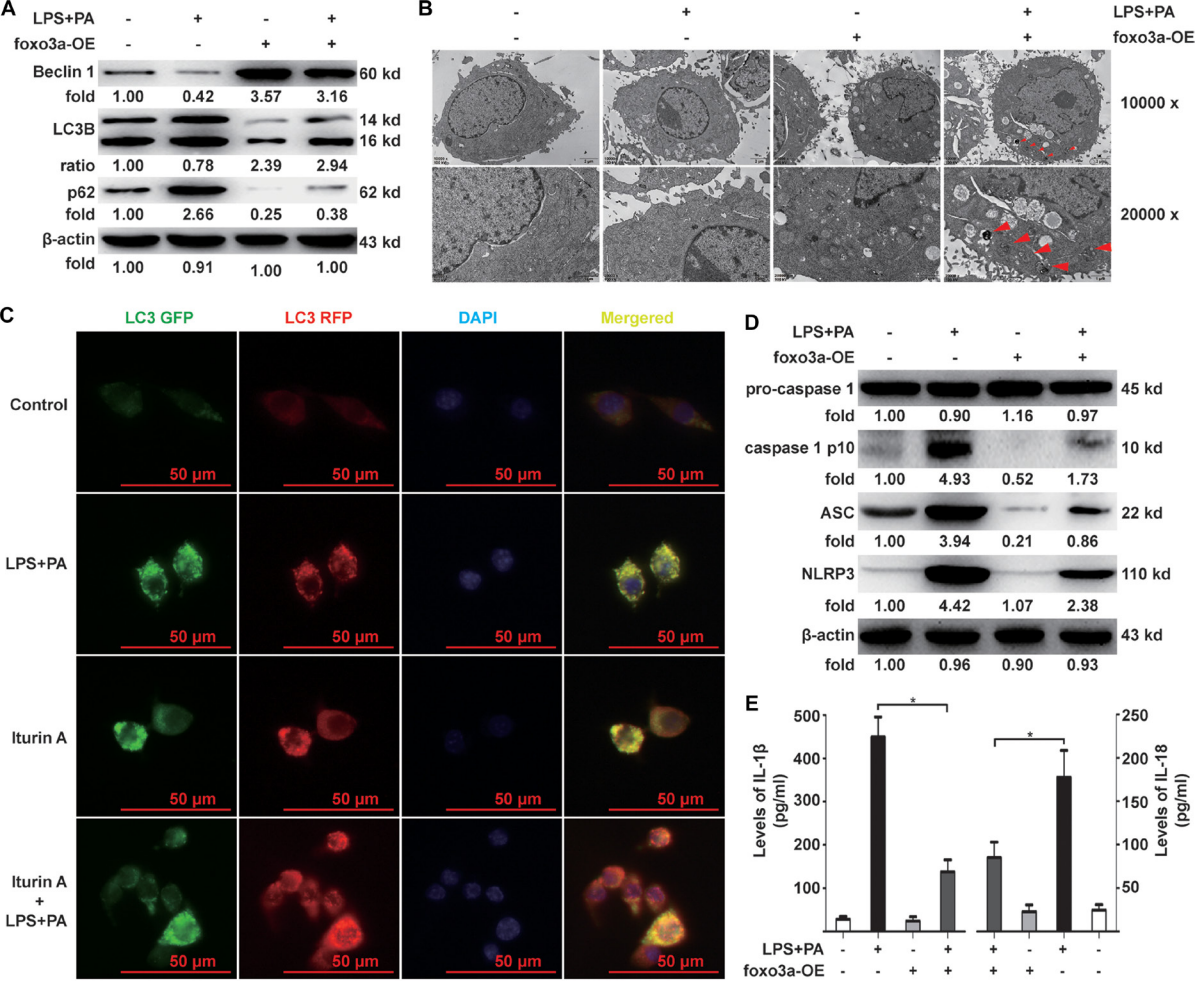
Effects of Foxo3a on autophagy flux and activation of the NLRP3 inflammasome in KCs (**A**) KCs were transfected with Foxo3a-OE plasmid for 48 h before stimulation with PA and LPS for 12 h. The ratio of LC3 II and I, and expression of Beclin 1 and p62, in the KCs were measured in KCs over-expressing Foxo3a. (**B**) Autophagosomes were observed by TME in KCs over-expressing Foxo3a (indicates autophagosomes ) (TME, 10000× and 20000×). (**C**) KCs were infected with mRFP-GFP-LC3-deficient adenovirus for 48 h followed by stimulation with PA and LPS for 12 h. mRFP-LC3 punctae in KCs treated with Iturin A (6 μM) for 1 h before the KCs were treated with PA and LPS were observed to be significantly more numerous compared to KCs treated with PA and LPS alone (FITC and TRITC, 400×). (**D**) The protein expression levels of the NLRP3 inflammasome and ACS, as well as caspase-1 separated from pro-caspase-1 in the KCs over-expressed Foxo3a were significantly lower than those KCs treated with PA and LPS alone. (**E**) The mRNA expression of IL-1β and IL-18 in KCs over-expressing Foxo3a were significantly lower than those KCs treated with PA and LPS alone. *p < 0.05.

KCs transfected with Foxo3a over-expression plasmid (Foxo3a-OE) shRNA (Foxo3a-shRNA, Supplementary Figure 4) were stimulated with PA and LPS for 12 h in order to observe changes in the autophagy flux and the activation of the NLRP3 inflammasome. The ratio of LC3 II and I, as well as the expression of Beclin 1, in KCs over-expressed Foxo3a were significantly higher than in KCs treated with PA and LPS alone (Figure 2A). Expression of p62 in the KCs over-expressing Foxo3a was significantly lower than in KCs treated with PA and LPS alone (Figure 2A). Additionally, autophagosomes were observed by TME in KCs over-expressing Foxo3a (Figure 2B). LC3 punctae were observed in the KCs treated with an agonist (Iturin A (6 μM) of Foxo3a after the KCs were treated with PA and LPS (Figure 2C). For NLRP3 inflammasome activation, the expression of IL-1β, IL-18, the NLRP3 inflammasome and ACS as well as caspase-1 separated from pro-caspase-1 in KCs over-expressing Foxo3a were all significantly lower than in KCs treated with PA and LPS alone (Figure 2D and 2E). In contrast, the expression of Beclin 1 in KCs with knock-down Foxo3a were significantly lower than in KCs treated with PA and LPS alone (Supplementary Figure 4A). The ratio of LC3 II and I as well as Expression of p62 with knock-down Foxo3a was significantly higher than in KCs treated with PA and LPS alone (Supplementary Figure 4A). For NLRP3 inflammasome activation, the expression of IL-1β, IL-18, the NLRP3 inflammasome and ACS as well as caspase-1 separated from pro-caspase-1 in KCs with knock-down of Foxo3a were all significantly higher than in those KCs treated with PA and LPS alone (Supplementary Figure 4B and 4C). Thus, the data have shown that autophagy flux is restored with the overexpression of Foxo3a, thereby dampening the activation of the NLRP3 inflammasome in KCs stimulated with PA and LPS. However, the patterns of Foxo3a restoration of the autophagy program were still unclear; therefore, the following investigation were carried out.

### Patterns of Foxo3a restoration of autophagy program

**Figure 3:**
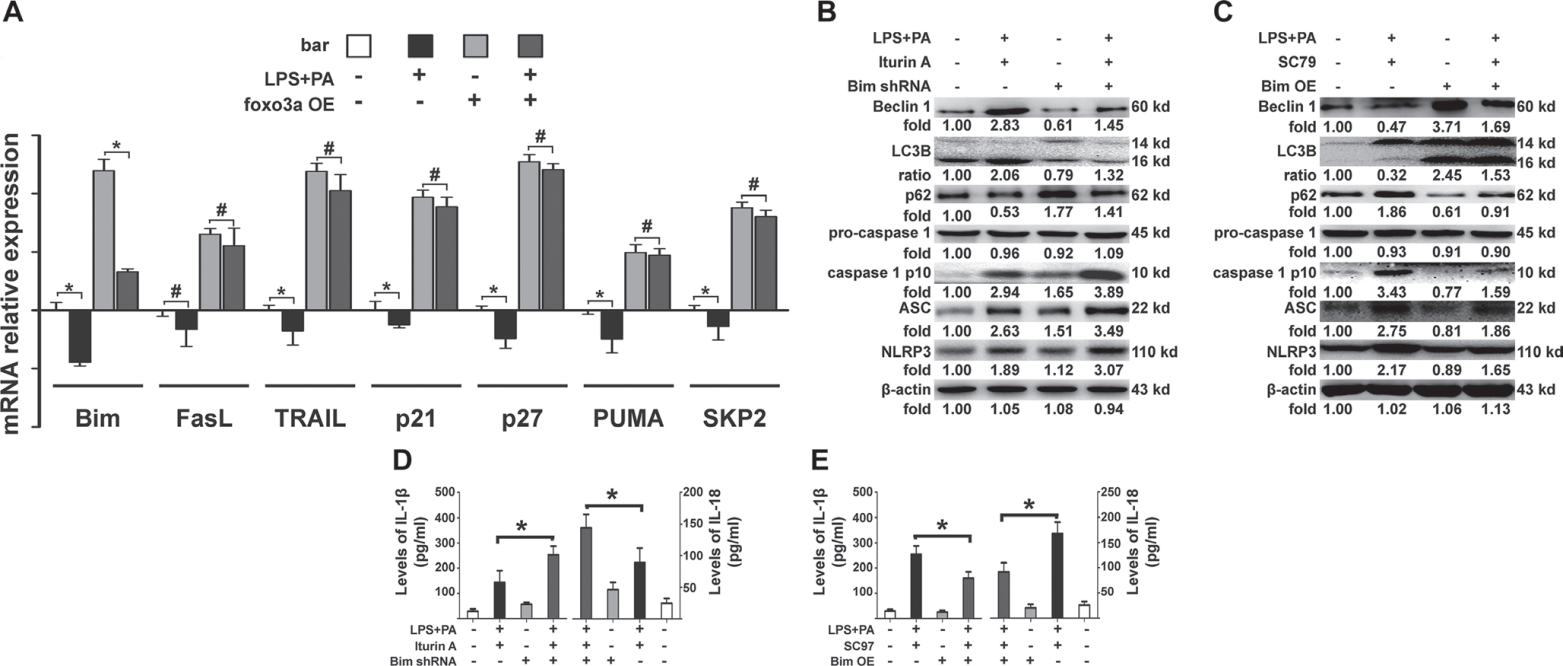
Patterns of Foxo3a restoration of the autophagy program (**A**) mRNA levels of Bim, FasL, TRAIL, p21, p27, PUMA and SKP2 were evaluated in KCs transfected with Foxo3a-OE plasmid and KCs stimulated with PA and LPS. Only the mRNA levels of Bim were dramatically down-regulated in KCs after stimulation with PA and LPS. *p < 0.05, ^#^*p* > 0.05. (**B**) The ratio of LC3 II and I, the expression of Beclin 1, p62, the NLRP3 inflammasome and ACS, as well as caspase-1 separated from pro-caspase-1 in KCs with Bim suppression before treatment with Iturin A (6 μM) and PA and LPS. (**C**) The ratio of LC3 II and I, and the expression of Beclin 1, p62, the NLRP3 inflammasome and ACS, as well as caspase-1 separated from pro-caspase-1, in KCs over-expressing Bim before treatment with SC97 (4 μg/ml) (S7863, Selleck, USA) (SC97 was dissolved in dimethylsulfoxide at room temperature) and PA and LPS. (**D**) The mRNA expression of IL-1β and IL-18 in KCs with Bim suppression before treatment with Iturin A (6 μM) and PA and LPS. *p < 0.05. (**E**) The mRNA expression of IL-1β and IL-18 in KCs over-expressing Bim before treatment with SC97 (4 μg/ml) and PA and LPS. *p < 0.05.

Various target genes that contain Foxo3a sequence-specific DNA binding sites were detected in KCs pretreated with an agonist or inhibitor of Foxo3a after KCs stimulation with PA and LPS in order to hunt for targets of Foxo3a. mRNA levels of Bim, FasL, TRAIL, p21, p27, PUMA and SKP2 were significantly up-regulated in the KCs transfected with a Foxo3a-OE plasmid (Figure 3A). Interestingly, only the mRNA levels of Bim were dramatically down-regulated in KCs after stimulation with PA and LPS (Figure 3A). We further detected changes in the autophagy flux in KCs with knocked-down Bim and altered Foxo3a conditions, before the KCs were treated with LPS and PA. The ratio of LC3 II and I as well as the expression of Beclin 1 in KCs with knocked-down Bim were significantly lower than in KCs treated with an agonist for Foxo3a and PA and LPS (Figure 3B). Expression of p62 in KCs with knocked-down Bim was significantly higher than in KCs treated with an agonist for Foxo3a and PA and LPS (Figure 3B). These results led to activation of the NLRP3 inflammasome pathway (Figure 3B and 3D). Conversely, the ratio of LC3 II and I as well as the expression of Beclin 1 in KCs over-expressing Bim were significantly higher than in KCs treated with an inhibitor (SC97 (4 μg/ml) for foxo3a and PA and LPS (Figure 3C). Expression of p62 in KCs over-expressing Bim was significantly lower than in KCs treated with an inhibitor for Foxo3a and PA and LPS (Figure 3C). These results led to attenuation of the NLRP3 inflammasome pathway (Figure 3C and 3E). Thus, these data have shown that autophagy flux was impaired by FFA and LPS *in vitro* via Foxo3a repression of the transcription of Bim, which promots the separation of Beclin 1 from the Dynein to initiate autophagy formation, as previously reported [25]. However, regulatory effects of Foxo3a in mice under HFD conditions were still a mystery, and we continued to explore in the following experiments.

### Effects of Foxo3a in mice fed with a HFD

**Figure 4:**
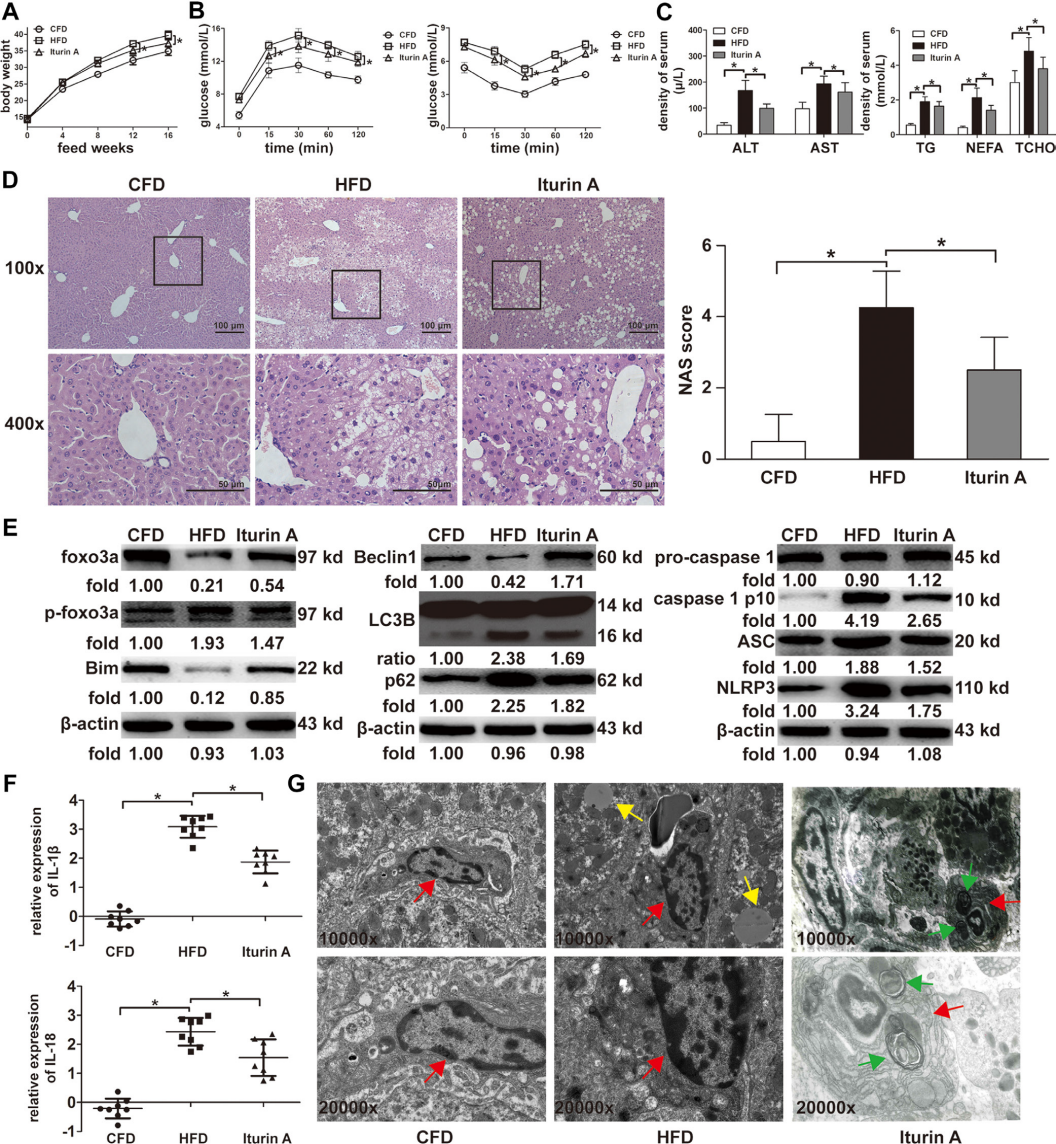
Effects of Foxo3a on mice feed with a HFD (**A**) Body weights in the Iturin A group were lighter compared to mice in the HFD group. (**B**) Glucose tolerance tests (left) in the Iturin A group were significantly better than for mice in the HFD group; insulin tolerance tests (right) in the Iturin A group were significantly better than for mice in the HFD group. (**C**) Serum levels of ALT, AST, TG, TCHO and NEFA were significantly lower than for mice in the HFD group. (**D**) Liver steatosis in the Iturin A group was more lighter compared to mice in the HFD group via H&E staining (100× and 400×); NAS scores in the Iturin A group were lower for those mice in the HFD group. (**E**) The ratio of LC3 II and I, the protein levels of Foxo3a, Bim, Beclin 1, the NLRP3 inflammasome and ACS, as well as caspase-1 separated from pro-caspase-1, in KCs isolated from mice in the Iturin A and HFD groups were evaluated via WB assay. (**F**) The mRNA expression of IL-1β and IL-18 in KCs isolated from mice in the Iturin A and HFD groups were evaluated using RT-PCR assay. *p < 0.05. (**G**) Autophagosomes of KCs were observed by TME in mice treated with Iturin A. (red arrow indicates KCs, yellow arrow indicates lipid droplets and green arrow indicates autophagosomes). (TME, 10000× and 20000×).

Mice were treated with Iturin A and fed with a HFD for 16 weeks in order to estimate the effects of Foxo3a in the NAFLD mice. Body weight, insulin and glucose tolerance test, levels of liver function, and severity of liver steatosis in the Iturin A group were better compared with mice in the HFD group (Figure 4A–4D). Protein levels of Foxo3a and Bim in KCs isolated from mice in the Iturin A group were significantly higher than in KCs from the HFD group (Figure 4E). Meanwhile, the expression of Beclin 1 in KCs isolated from mice in the Iturin A group were significantly higher than for KCs from the HFD group (Figure 4E). The ratio of LC3 II and I as well as the expression of P62 in KCs isolated isolated from mice in the Iturin A group was significantly lower than in KCs from the HFD group (Figure 4E). For NLRP3 inflammasome activation, the expression of IL-1β, IL-18, the NLRP3 inflammasome and ACS as well as caspase-1 separated from pro-caspase-1 in KCs isolated from mice in the Iturin A group were significantly lower than in KCs from the HFD group (Figure 4E and 4F). Furthermore, autophagosomes of KCs were observed by TME in mice treated with Iturin A (Figure 4G). Thus, the data have shown that the promotion of transcription of Bim by Foxo3a protects mice from a HFD via inhibiting the activation of the NLRP3 inflammasome by facilitating autophagy flux in KCs.

## DISCUSSION

Accumulation of excessive and toxic lipids in the liver causes persistent, low-level inflammation and ROS [26]. KCs, the resident macrophages in liver tissue, are first triggered and initiated an innate immune response [4–5]. Additionally, the innate function of KCs such as phagocytosis and autophagy, are impaired by FFA [15, 27]. Impairing autophagy in KCs triggers the KCs to M1 polarization, which increases the immune response in obese mice [15]. Previous studies have asserted that autophagy flux attenuates activation of the NLRP3 inflammasome pathway in macrophages [18, 28]. Thus, restoration of autophagy flux in KCs may theoretically dampen the damage caused by the NLRP3 inflammasome.

Autophagy is induced by many molecules in different cells under relevant conditions. Chen et al. have asserted that Dihydromyricetin promotes autophagy-related genes and suppresses liver ischemia and reperfusion-induced apoptosis via elevating expression and nuclear translocation [29]. Liu et al. have confirmed that osteopontin induced autophagy promotes chemoresistance in hepatocellular carcinoma cells via sustaining Foxo3a stability [30]. Consistently, the data in this study have shown that autophagy flux previously impaired by PA and LPS can be restored via overexpression of Foxo3a, which subsequently alleviates activation of the NLRP3 inflammasome. Conversely, Shin et al. have asserted that nutritional deprivation promotes autophagy flux via Foxo3a phosphorylation nuclear exportation, which represses transcription of SKP2 in mouse embryonic fibroblasts [19]. Collectively, Foxo3a induces autophagy flux via altering its transcriptional activity under different stress conditions; there are the main ways in which Foxo3a participates in the regulation of autophagy. In this study, the transcriptional activity of Foxo3a was inhibited by PI3K/AKT activation in KCs treated with PA and LPS, leading to the blockage of autophagy flux.

Foxo3a rapidly mounts an autophagy program under stressful conditions via maintaining gene transcription [31]. Foxo3a is involved in autophagy programs via inducing downstream target gene expression, such as manganese superoxide dismutases, catalase, Bim, Noxa, FasL, TRAIL, p21, p27, PUMA and SKP2 [32]. These genes regulate to ROS, apoptosis and autophagy. In this study, only Bim was dramatically decreased in KCs treated with PA and LPS, which is consistent with the down-regulation of Foxo3a in the nucleus. Bim, a pro-apoptotic factor and a member of the Bcl-2 family, integrates with Dynein at the LC8 light chain at rest and is inactive [33]. Bim and the LC8 are separated from the complex and become activated under stress conditions [33]. Additionally, Beclin 1 is anchored to the BH3 region of Bim and connected with LC3 when the autophagy program is inhibited [25]. Beclin 1 is released into the cytoplasm to initiate the autophagy program as soon as Bim is activated through phosphorylation [25]. Furthermore, Dynein, once separated from Bim, also participates in the autophagy program from autophagosome to autolysosome [34]. Thus, the Foxo3a-dependent promotion of Bim transcription protects mice from HFD.

In conclusion, Foxo3a is depressed in the nucleus while autophagy is impaired, and NLRP3 inflammasome is activated in KCs. Over-expression of Foxo3a restores autophagy flux and attenuates activation of the NLRP3 inflammasome via promoting the transcription of Bim, which induces Beclin 1 separation from LC8 and initiates the autophagy program. This is another pattern of Foxo3a regulation of autophagy, suggesting a potential therapeutic target in NAFLD and other insulin resistance-related diseases.

## MATERIALS AND METHODS

### Isolation and treatment of murine KCs

Isolation of KCs was performed according to our previously published methods [35]. The purity of isolated KCs was greater than 70%. Purified KCs were treated with increasing doses of PA (P5585, Sigma, USA) (0–0.64 mM) for 12 h after pretreatment with LPS (L5293, Sigma, USA) for 1 h. PA was dissolved in 0.01mol/L sodium hydroxide at 70°C for 30 min, then mixed with 30% BSA at 70°C before sterilization and used it directly after preparation. LPS was dissolved in 0.01 M sterile phosphatic buffer solution (PBS) at room temperature. The phosphorylation levels of Foxo3a, as well as the supernatant levels of IL-1β and IL-18, were measuredd to seek an optimal concentration for later experiments.

### Transfection assay

foxo3a-OE plasmid and Foxo3a-shRNA, Bim-OE plasmid and Bim-shRNA, as well as mRFP-GFP-LC3 and GFP-LC3-deficient adenoviruses, were purchased from Genecopoeia Inc. (USA). OE plasmids and shRNA were transfected with Lipofectamine^®^3000 into KCs for 48 h, and the efficacy effect was greater than 50% (Supplementary Figure 1A and 1B). KCs were infected with mRFP-GFP-LC3 and GFP-LC3-deficient adenoviruses according to the manufacturer instruction. LC3 punctae were observed by fluorescence microscopy after stimulation with PA and LPS. The excitation wavelength for RFP-LC3 was 550–590 nm, for GFP-LC3 was 395–495 nm, and for DAPI was 358–360 nm

### Transmission electron microscopy

KCs were collected, fixed and solidified according to routine methods. After drying naturally, the samples were adhered to the slices. KCs in the slices were observed with transmission electron microscope (TEM).

### Enzyme-linked immunosorbent assays

The supernatant levels of IL-1β (EK0394, Boster, China) and IL-18 (C507380, Sangon Biotech, China) were quantified using commercially available ELISA kits. All the operations were in strict accordance with the manufacturer's instructions.

### Western blotting analysis

Protein was extracted from KCs (5 × 10^6^) with RIPA lysis buffer (P0013B, Beyotime, China) and quantified using the BCA protein quantitative kit (P0009, Beyotime, China). The protein samples were electrophoresed in 8–12% sodium dodecyl-sulfate polyacrylamide gels and trans-blotted onto polyvinylidene fluoride (PVDF) membranes. After binding with primary antibodies, the PVDF membranes were visualized using the Chemico-EQ system (Bio-Rad, USA). The value of the targeted protein bands was detected using Image Lab 3 software (Bio-Rad, the USA) and normalized against β-actin. The primary antibodies are showed at Supplementary Figure 2.

### RT-PCR analysis

Total RNA of KCs (5 × 10^6^) was extracted using Trizol buffer (R0016, Beyotime, China). The total RNA samples were reverse transcribed into cDNA strictly following the protocol of the Primescript™ RT reagent kit with the gDNA Eraser (RR047A, Takara, Japan). PCR was conducted using the SYBR premix Ex Taq II kit (RR820A, Takara, Japan). The relative expression of the targeted gene was determined using the –(delta delta C(T)) method after normalizing against the β-actin gene. The primers for the targeted genes are shown in Supplementary Figure 2.

### Animals

Pathogen-free C57BL/6 mice (male, 13–15 g, 4 weeks old) were obtained from the Experimental Animal Center of Chongqing Medical University, Chongqing, China. Mice were allowed free access to sterile water and food with good human care. All protocols related to animals were approved by the committee on ethics of the Chongqing medical university and according to the National Institutes of Health Guidelines.

### Experimental groups

A total of 24 mice were randomly divided into the following 3 groups: a coarse food diet group (CFD group), where mice were fed with standard laboratory chow diet; a high fat diet group (HFD group), where mice were fed with a high fat diet containing 24% protein, 41% carbohydrate and 24% fat (D12451, ResearchDiets, USA) for 16 weeks; and a high fat diet combined with Iturin A treatment group (Iturin A group), where mice were fed with a high fat diet and received an oral administration of Iturin A (5 mg/kg) for 16 weeks in alternative days. Iturin A was purchased from Sigma-Aldrich Co. LLC. (I1774, USA) and dissolved in ethanol at room temperature. The safe and effective dose ranges of Iturin A were previously determined [36].

Mice in each group were sacrificed at 16 weeks. Serum samples were collected for examination of liver function. Liver tissue samples were collected for separation of KCs, WB analysis, histology and Oil Red staining analysis.

### Glucose and insulin tolerance tests

For glucose tolerance tests, mice in each group were fasted for 8 h and injected intraperitoneally with glucose (1.5 g/kg) diluted with normal saline after feeding at 16 weeks. For insulin tolerance tests, mice in each group were fasted for 4 h and injected intraperitoneally with insulin (0.7 IU/kg). Blood was collected from the caudal vein at 0, 15, 30, 60 and 120 min after glucose or insulin injection. Plasma glucose was detected by a portable glucometer (One Touch Ultra, Johnson, USA).

### Liver function examination

The levels of alanine aminotransferase (ALT), aspartate amino transferase (AST), triglycerides (TG), total cholesterol (TCHO) and non-esterified fatty acids (NEFA) were detected by an automatic biochemical analyzer (Beckman, the USA).

### Histology analysis

Liver tissue was fixed using 4% paraformaldehyde at 37°C for 48 h before being embedded in paraffin. The paraffin samples were cut into 3- to 5-μm sections, followed by dewaxing and hydration. For histology analysis, the sections were stained using hematoxylin and eosin. Liver histology samples were scored for an NAFLD activity score (NAS) by three experienced pathologists according to the Kleiner Scoring System [37].

### Statistical analysis

All data were expressed as the mean ± standard deviation (x ± s) and analyzed using SPSS18.0 software (USA). The comparison of multiple groups was performed using one-way ANOVA, and two independent samples were analyzed using the Student's *T-test*. A *p value* less than 0.05 was set as a significant difference.

## SUPPLEMENTARY MATERIALS FIGURES AND TABLES



## Abbreviations

ALT: alanine aminotransferase
AMPK: AMP-activated protein kinase
ASC: apoptosis associated speck-like protein
AST: aspartate amino transferase
CFD: coarse food diet
DAMPs: danger-associated molecular patterns
foxo3a: fork O3A protein
HFD: high fat diet
IκB-α: inhibitor of NF-κB α
IL: interleukin
KCs: Kupffer cells
LPS: lipopolysaccharide
mtDNA: and mitochondrial DNA
MYD88: myeloid differentiation molecular 88
NALFD: non-alcoholic fatty liver disease
NAS: NAFLD activity score
NEFA: nonesterified fatty acid
NF-κb: nuclear factor κb
NLRP3: nucleotide oligomerization domain (NOD)-like receptor family pyrin domain containing 3 (NLRP3)
OE: over-expression plasmid
PA: palmitic acid
PAMPs: pathogen-associated molecular patterns
PI3K: phosphatidyl inositol 3-kinas
PVDF: polyvinylidene fluoride
ROS: reactive oxygen species
SIRT: silent information regulator
SKP2: S phase kinase-associated protein 2
TCHO: total cholesterol
TEM: transmission electron microscope
TG: triglyceride
TLR4: Toll-like receptor 4
