# Supplementary material for: Foxo3a-dependent Bim transcription protects mice from a high fat diet via inhibition of activation of the NLRP3 inflammasome by facilitating autophagy flux in Kupffer cells
Source: Oncotarget. 2017 Mar 6;8(21):34258–67. doi: 10.18632/oncotarget.15946 (PMC5470965; doi:10.18632/oncotarget.15946)
Supplement: Supplementary file 1 [file oncotarget-08-34258-s001.pdf]

Foxo3a-dependent Bim transcription protects mice from a high fat diet via inhibition of activation of the NLRP3 inflammasome by facilitating autophagy flux in Kupffer cells

Supplementary Materials

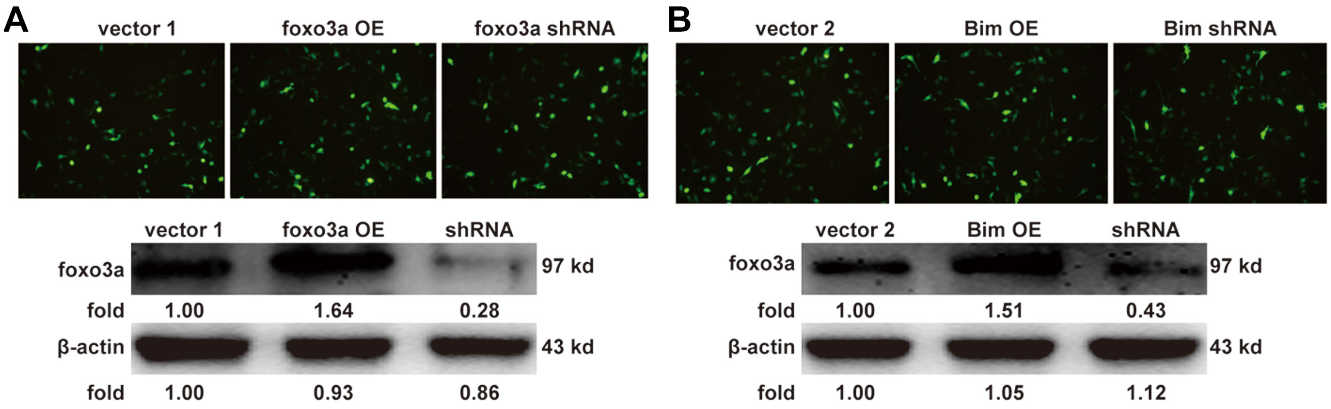

Supplementary Figure 1: Transfection of plasmids in KCs *in vitro*. (**A**) Expression of green fluorescent protein and Foxo3a in KCs after transfection with Foxo3a-OE and shRNA plasmid were detected by fluorescence microscope and WB assay, respectively. The expression of green fluorescent protein and Bim in KCs after transfection with Bim-OE plasmid and Bim-shRNA plasmid were detected by fluorescence microscope and WB assay, respectively. (FITC, 200×)..

1 Antibodies and dilution ratio

Rabbit anti-foxo3a antibody (12829, CST, the USA), 1:1000;
Rabbit anti-p-foxo3a antibody (9466, CST, the USA), 1:1000;
Rabbit anti-Bim antibody (ab32158, Abcam, the USA), 1:1000;
Rabbit anti-Beclin1 antibody (ab207612, Abcam, the USA), 1:1000;
Rabbit anti-LC3B antibody (2775, CST, the USA), 1:1000;
Rabbit anti-p62 antibody (5114, CST, the USA), 1:1000;
Rabbit anti-caspase 1 antibody (ab108362, Abcam, the USA), 1:5000;
Mouse anti-caspase 1 p10 antibody (sc-514, Santa cruz, the USA), 1:200;
Rabbit anti-ASC antibody (67824, CST, the USA), 1:1000;
Rabbit anti-NLRP3 antibody (15101, CST, the USA), 1:1000;
Rabbit anti-AMPKα antibody (5831, CST, the USA), 1:1000;
Rabbit anti-p-AMPKα antibody (2535, CST, the USA), 1:1000;
Rabbit anti-AKT antibody (4685, CST, the USA), 1:1000;
Rabbit anti-p-AKT antibody (4058, CST, the USA), 1:1000;
Rabbit anti-PI3K antibody (4257, CST, the USA), 1:1000;
Rabbit anti-p-PI3K antibody (4228, CST, the USA), 1:1000;
Mouse anti-β-actin antibody (BM0627, Boster, China), 1:200;
Goat anti-rabbit IgG HRP antibody (BA1055, Boster, China), 1:2000;
Goat anti-mouse IgG HRP antibody (BA1051, Boster, China), 1:2000;

2 Primers for targeted genes

Targeted genes	Primers	Length (bp)
IL-1β	Forward: 5′-GCAACTGTTCCTGAACTCAACT-3′<br>Reverse: 5′-ATCTTTTGGGGTCCGTCAACT-3′	89
IL-18	Forward: 5′-GACTCTTGCGTCAACTTCAAGG-3′<br>Reverse: 5′-CAGGCTGTCTTTTGTCAACGA-3′	169
β-actin	Forward: 5′-CCTCTATGCCAACACAGTGC-3′<br>Reverse: 5′-GTACTCCTGCTTGCTGATCC-3′	211

Supplementary Figure 2: Antibody dilution ratios and primers for targeted genes.

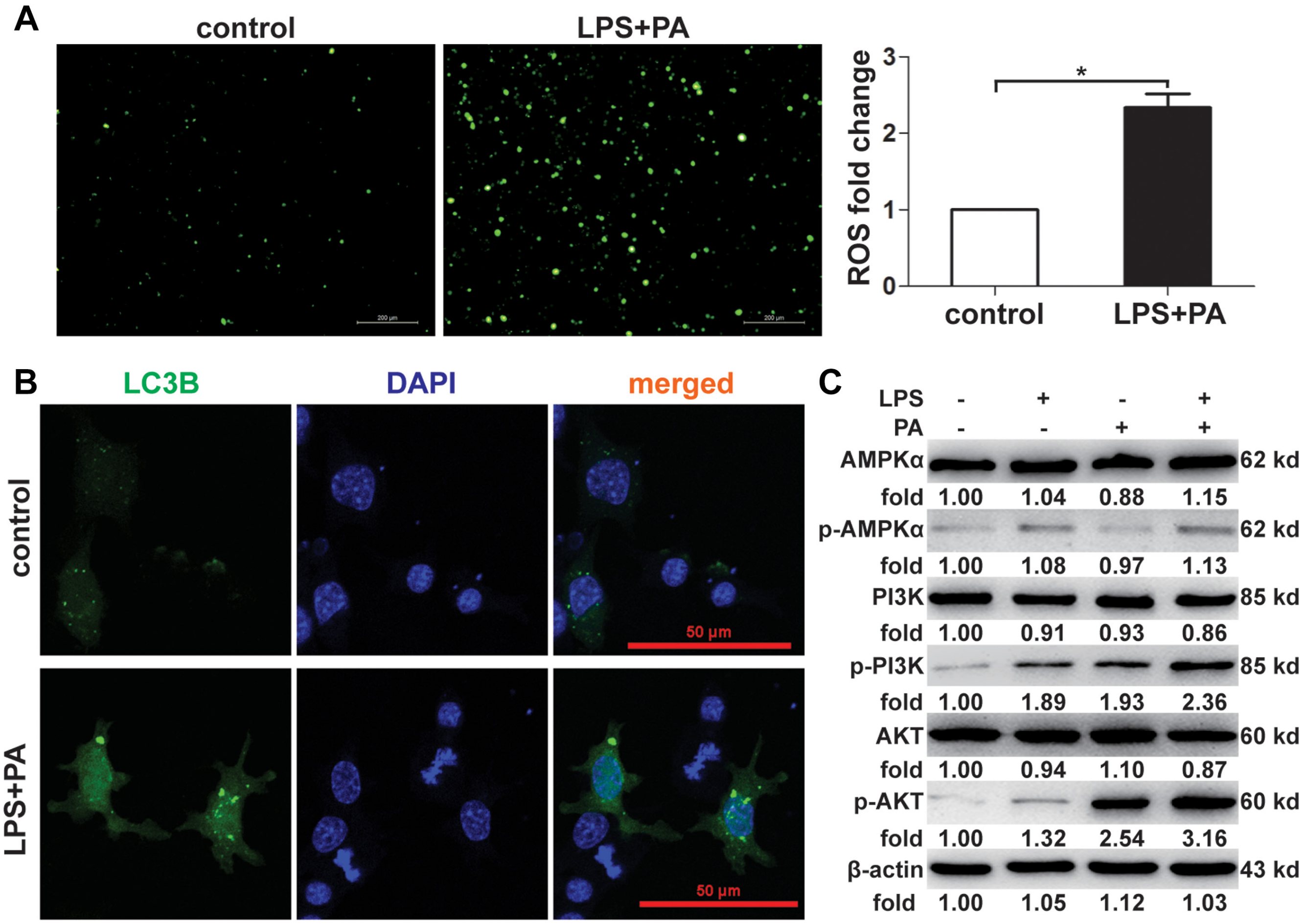

Supplementary Figure 3: KCs were treated with PA (0.5 mM) for 12 h after stimulating with LPS (100 ng/ml) for 1 h. (**A**) ROS were increased in KCs treated with PA and LPS (FITC, 100×). (**B**) LC3 punctaee were increased in KCs treated with PA and LPS (FITC, 400×). (**C**) The phosphorylation levels of PI3K/AKT were significantly higher in KCs treated with LPS and PA than in KCs treated with PA or LPS alone, whereas phosphorylation levels of AMPK were unchanged among the four groups.

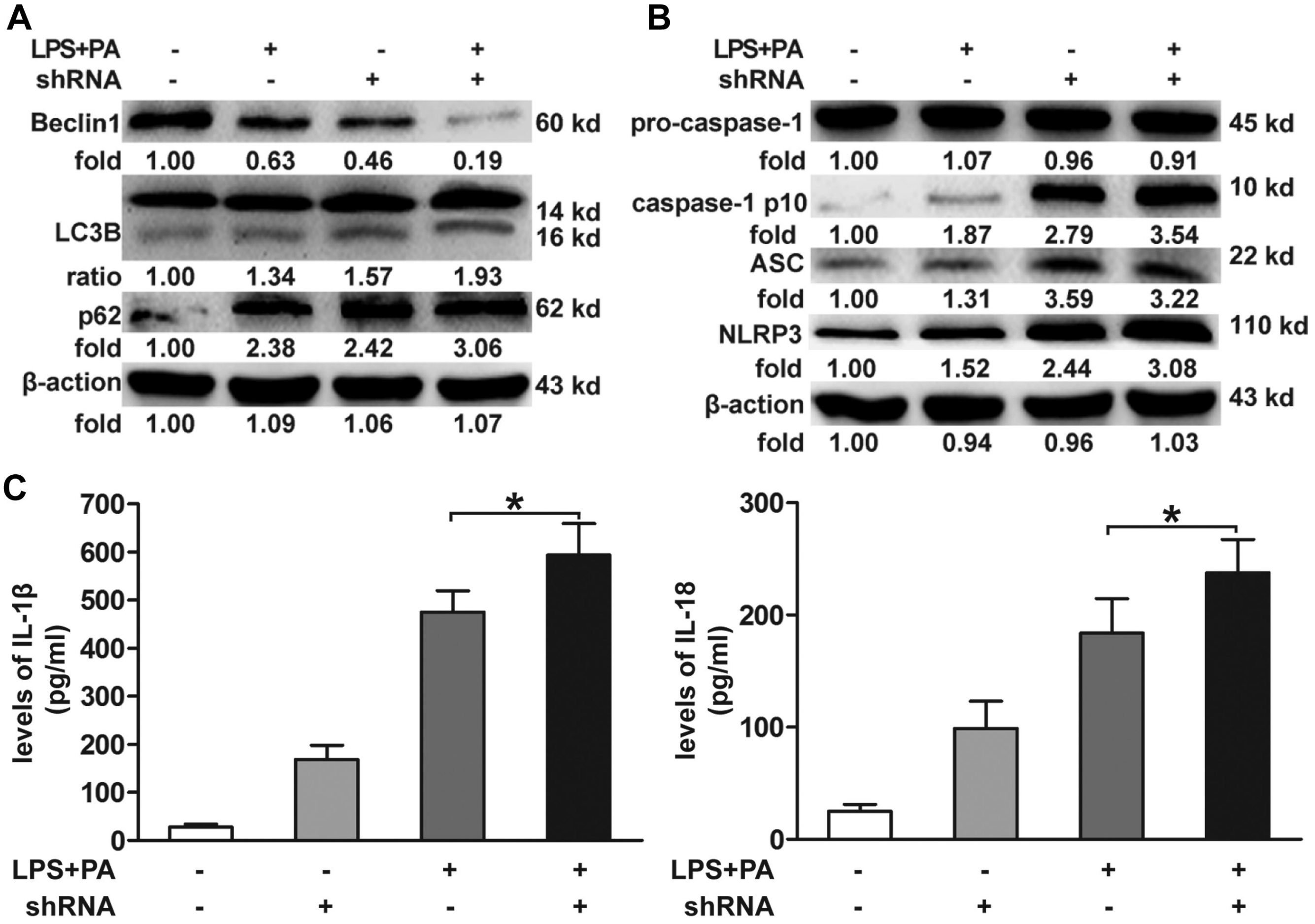

Supplementary Figure 4: KCs were transfected with a Foxo3a-shRNA plasmid for 48 h before stimulating with PA and LPS for 12 h. (**A**) The ratio of LC3 II and I in KCs, as well as the protein levels of Bim and Beclin1\ were detected by WB assay. (**B**) Protein levels of the NLRP3 inflammasome and ACS in KCs, as well as levels of caspase-1 separated from pro-caspase-1, were detected by WB assay. (**C**) mRNA levels of IL-1β and IL-18 in KCs were detected by RT-PCR assay. *$p < 0.05$.
